# FaceSync: Open source framework for recording facial expressions with head-mounted cameras

**DOI:** 10.12688/f1000research.18187.1

**Published:** 2019-05-21

**Authors:** Jin Hyun Cheong, Sawyer Brooks, Luke J. Chang

**Affiliations:** 1 Psychological and Brain Sciences, Dartmouth College, Hanover, NH, 03755, USA; 2 Department of Neuroscience, Oberlin College, Oberlin, Ohio, 44074, USA

**Keywords:** facial expressions, affective computing, Python toolbox, head-mounted camera, synchronization

## Abstract

Advances in computer vision and machine learning algorithms have enabled researchers to extract facial expression data from face video recordings with greater ease and speed than standard manual coding methods, which has led to a dramatic increase in the pace of facial expression research. However, there are many limitations in recording facial expressions in laboratory settings.  Conventional video recording setups using webcams, tripod-mounted cameras, or pan-tilt-zoom cameras require making compromises between cost, reliability, and flexibility. As an alternative, we propose the use of a mobile head-mounted camera that can be easily constructed from our open-source instructions and blueprints at a fraction of the cost of conventional setups. The head-mounted camera framework is supported by the open source Python toolbox FaceSync, which provides an automated method for synchronizing videos. We provide four proof-of-concept studies demonstrating the benefits of this recording system in reliably measuring and analyzing facial expressions in diverse experimental setups, including group interaction experiments.

## Introduction

Facial expressions provide rich information about how a person is feeling, what they are thinking, and how they might act ( Russell & Fernández-Dols, 1997). Facial expressions captured the interest of early theorists ( Darwin, 1872; James, 1884) and remain a popular method for noninvasively studying behavioral displays of emotions. Pioneering work by Paul Ekman established the Facial Action Coding System (FACS; Ekman & Oster, 1979), which provided a reliable coding system of different facial muscles referred to as action units (AUs) and allowed facial expressions to be compared across people and cultures ( Matsumoto & Ekman, 1989; Matsumoto *et al.*, 2009).

Extracting facial expression information through FACS coding, however, can be a labor intensive and time-consuming process. Becoming a certified FACS coder not only requires 100 hours of training ( “Paul Ekman Group,” 2017) but even a well-trained coder may need over an hour to code a single minute of video ( Cohn *et al.*, 2007). In addition, manual coding inevitably exposes the data to human errors in coding or biases, therefore requiring researchers to collect ratings from more than one coder, further complicating the process. 

As an alternative to manual FACS coding, automated measurements of facial expressions can substantially reduce the amount of time required to extract facial expression information. One technique, known as facial electromyography (fEMG), measures the electrical impulses associated with facial muscle movements. With fEMG, researchers can continuously measure the activity from muscle groups associated with facial AUs, such as the zygomaticus major and corrugator supercilii muscles, at a high sampling rate. However, fEMG requires a separate electrode for each facial muscle group, which means that only one or two muscle groups are recorded simultaneously in practice ( Fridlund & Cacioppo, 1986; Wolf, 2015). Even so, the recordings may include signals from not only the target muscle but also overlapping or nearby muscles making it difficult to distinguish the precise activity of the target muscles ( Cohn & Ekman, 2005). Moreover, this technique does not scale well to recording multiple participants interacting in a social experiment as recordings can be sensitive to movement artifacts and having wires attached to one’s face can be unnatural and obtrusive.

Automated extraction of facial expression information from face video recordings have emerged as a promising alternative that offers quick, continuous, and simultaneous measurement of multiple facial muscle movements without manual coding. Advances in computer vision and machine-learning techniques (e.g., kernel methods, deep learning) and large-scale data collection have facilitated the development of models that learn to transform pixels from videos into predictions of facial AUs and emotional facial expressions ( Amos *et al.*, 2016; Littlewort *et al.*, 2011; Michel & Kaliouby, 2003; Susskind *et al.*, 2007). Consequently, this has facilitated an explosion in scientific articles related to facial expression analysis with a sixfold increase over the past decade. ^1^

This automated approach has offered much insight into human behavior. Automated extraction of facial expressions has been used to predict a wide range of behaviors including task engagement ( Whitehill *et al.*, 2014), automobile accidents ( Ahn *et al.*, 2010), effectiveness of advertisements ( McDuff *et al.*, 2015), and online purchase behaviors ( Ahn *et al.*, 2008). Cultural differences in facial behavior have also been examined at a larger scale spanning more than 31 countries ( McDuff *et al.*, 2017) as well as sex differences in smiling ( McDuff *et al.*, 2017). Facial expressions have also shown promise in clinical settings to quantify symptom severity in neuropsychiatric disorders such as Schizophrenia and Parkinson’s disease ( Bandini *et al.*, 2017; Hamm *et al.*, 2011) and depression ( Girard *et al.*, 2015), and also for detecting evidence of malingering pain symptoms ( Bartlett *et al.*, 2014).

**Figure 1.**
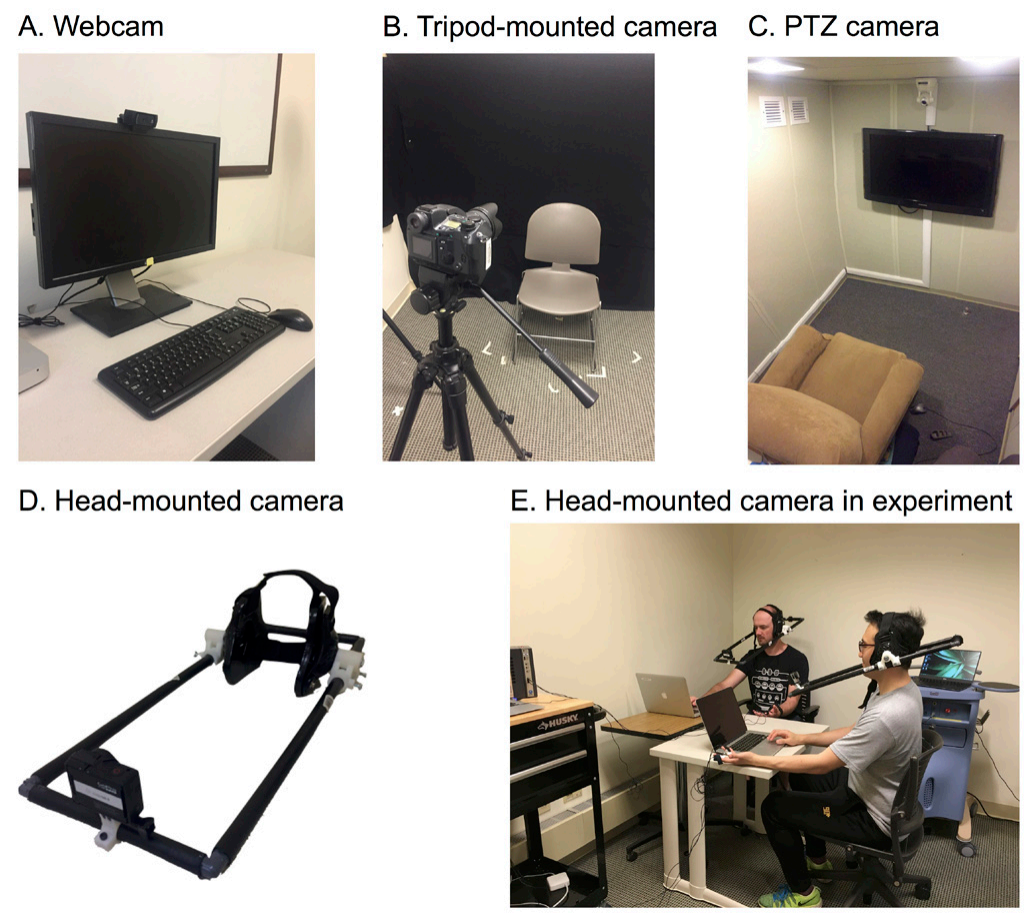
Different recording setups. ( **A**). Webcam setup with a camera positioned above the monitor. ( **B**). Tripod-mounted camera setup placed facing the subject. ( **C**). PTZ camera setup with the camera installed above the TV screen in a dedicated room. ( **D**). Head-mounted camera built according to build instructions in the *Underlying data*. ( **E**). Use of head-mounted camera in social interaction experiment.

The *acquisition* of high temporal and spatial resolution of facial expressions in laboratory environments, however, has remained challenging. Popular solutions such as webcams, tripod-mounted cameras, and pan-tilt-zoom (PTZ) cameras ( Figure 1A–C) require compromising between cost, flexibility, and reliability. In this article, we demonstrate the feasibility of head-mounted video cameras as an alternative to standard recording setups. We provide step-by-step instructions on how to build affordable head mounts using readily available materials and minimal technical expertise. We demonstrate how the head-mounted camera can provide reliable recordings that is invariant to head-rotation and can be flexibly used in a variety of experimental settings such as stimulus based tasks, natural viewing of videos, and social interactions. We also introduce the FaceSync toolbox which can be used in conjunction with head-mounted cameras to automatically synchronize videos in time based on audio. Overall, we provide a unique solution for recording facial expressions that is affordable, adaptable to different experimental setups, and reliable in recording an unobstructed view of the face.

### Selecting a recording method

When choosing a framework for recording videos of facial behavior in lab settings, researchers must consider a variety of factors including affordability, adaptability to different experimental settings, integration with other devices, and recording reliability. In this section we survey and summarize the strengths and shortcomings of popular setups including webcams, tripod-mounted cameras, and PTZ cameras ( Table 1).

**Table 1.** Relative comparison of facial expression recording methods.

	Sampling resolution	Adaptability to different experimental settings	Ease of integration with other devices	Robustness to face rotation or obstruction	Subtlety of recording device	Affordability
fEMG	High	Low	Medium	High	Low	Low
Webcams	Low	Low	High	Low	High	High
Tripod-mounted cameras	Medium	Medium	Low	Low	Low	Medium
PTZ cameras	Medium	Low	Low	Low	High	Low
Head-mounted cameras	High	High	Medium	Medium	Low	High

The most readily available and easy-to-implement option is to record from computer webcams. External webcams with good image resolution can cost about $100 but most modern laptops and computers come with pre-installed webcams integrated on top of the screen providing a low profile setting less likely to capture the attention of participants. Webcams are effective for event based experiments in which facial expressions can be recorded using the same computer hosting the task. Webcams can be triggered and controlled via programming languages providing a scalable solution for recording social interactions through video call setups. Such interactions, however, may not provide the same experience as live face-to-face interactions ( Sherman *et al.*, 2013; Shin *et al.*, 2017). Moreover, integrated webcams can be limited in temporal resolution as they rely on shared computing resources and use variable frame rates between 24 to 30 frames per second (fps) to optimize the use of computer resources. In addition, the fixed position of webcams and the distance between the camera and the face allow bodily movements touching the face, head rotations, or out-of-plane head motions to cause difficulties in capturing and extracting facial expressions from the videos. Therefore, it is difficult to consider webcams as a robust and reliable solution to recording facial expressions despite being easy to use and cost-effective.

Tripod-mounted cameras cost around $1,000 for production-quality camcorders and can provide high-resolution recordings at faster frame rates. Tripod-mounted cameras can be manually moved or adjusted by the experimenter to account for subject movements if the experimenter can be present during the experiment at the cost of increased conspicuousness. Tripod-mounted cameras can be easily installed and removed to accommodate different experimental settings allowing for flexibility in experimental setups. They can moved to different experimental environments and adjusted to different heights and angles to best capture facial behaviors. Scalability, however, is limited as adding additional cameras remains expensive and synchronizing across multiple cameras can be challenging as time-code or TTL (transistor-transistor-logic) pulse triggering capable camcorders are often more expensive.

The PTZ camera setup provides researchers centralized control over cameras that can be rotated or zoomed to account for subject movement. PTZ camera setups require a dedicated experiment room with cameras installed and an adjacent console room where experimenters can monitor incoming video feeds and control the camera. Central management of cameras can facilitate integration with other softwares or triggering the cameras to record simultaneously. The installation of cameras to corners of ceilings distant from the participant allows cameras to be less conspicuous. However, this forces participants to stand or sit in particular locations in the room and renders the setup particularly susceptible to head rotations or occlusions of the face from body gestures unless multiple cameras are installed. It is the least flexible option because changing camera locations or installing additional cameras would require additional construction. The PTZ camera setup can therefore be the best option to minimize participants’ attention to the camera but at the cost of increased possibility of artifacts and reduced adaptability to other experimental setups.

Overall, webcams, tripod-mounted cameras, and PTZ cameras do not provide an optimal solution for recording facial expressions. They commonly suffer data loss due to out-of-plane head motions and head rotations ( Cohn & Sayette, 2010; Lucey *et al.*, 2011; Werner *et al.*, 2013), although developing algorithms robust to partial face occlusions is an active area of research ( Zhang *et al.*, 2018). Another common challenge pertains to the temporal precision in the alignment of simultaneous recordings between cameras and to the stimuli. In the next section, we propose head-mounted cameras as an affordable, scalable, and flexible solution that can provide reliable recordings and can be easily synchronized with experimental stimuli and across recordings from multiple cameras.

## Head-mounted cameras

A head-mounted camera recording system ( Figure 1D) provides a unique solution to the limitations of the surveyed methods. It is a highly adaptive system that can be used for different experimental setups ranging from computer-based tasks to multi-person social interaction experiments ( Figure 1E). The head-mounted camera consists of a single camera attached to the head of the participant using lightweight head-gear. This setup removes the impact of head rotation or body movements obstructing the view of the face leading to face detection failure and increases reliability. However, as a result, it cannot detect bodily movements and gestures, unless additional cameras are installed, or head orientation information, unless additional gyro sensors are attached. It is minimally cumbersome other than the weight of the gear and protrusion from the head-gear, and it can be positioned below the line of sight of subjects, allowing the wearer to view a monitor in computer based tasks or make eye contact and track others’ facial expressions in social interaction tasks.

Commercial head-mounted cameras are often used in motion capture studios and remain expensive with costs ranging from $2,500 - $20,000 for a complete camera and mount setup ^2^. However, assembling a head-mounted camera setup in the lab can be an affordable alternative option that requires minimal engineering expertise or expensive equipment. Action cameras, such as the GoPro are well-suited for this purpose as they are inexpensive ($150 - $400), small in size, and lightweight. We provide step-by-step assembly instructions for building a head-mount for GoPro cameras in the Supplementary Information (see *Underlying data)* ( Cheong *et al.*, 2019) along with a parts list and blueprint files to 3D print other parts. This allows researchers to easily construct their own head-mounted camera setup for less than $700 (including the camera) ^3^.

### Synchronizing videos to stimuli using audio features

All video recording devices require a method to temporally align the video recordings to the experimental task. As mentioned earlier, some devices such as webcams or PTZ cameras can be controlled or triggered from the experiment computer to start and stop recording during the paradigm. Camera setups that are not directly connected to the experiment computer, including head-mounted devices, require an alternative method that is accurate and efficient for aligning the videos to experimental events.

The traditional ‘clap’ method used in the film industry uses a sharp, loud sound at the beginning of the clip that allows multiple videos to be aligned to the resulting spike in the audio waveform. This audio-based synchronization method usually requires opening each video for manual inspection of the sound waveform and incrementally shifting the audio until the echo, which indicates phase misalignment, is eliminated. Humans are highly accurate in detecting and distinguishing audio offsets down to several milliseconds (ms), but manually synchronizing each video is labor intensive and can introduce unsystematic noise in the alignment ( Litovsky *et al.*, 1999; Shrstha *et al.*, 2007).

**Figure 2.**
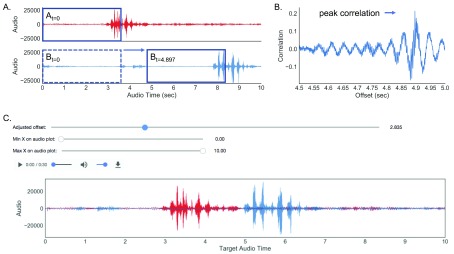
Schematic of automatic and manual audio alignment method. ( **A**, **B**) Portion of audio is selected from target audio (red waveform top panel) which is compared to a portion of the sample audio selected in a sliding temporal window (blue waveform bottom panel). Correlation similarity is calculated at each window and the offset is determined to be the temporal window that maximizes similarity between the two audios. ( **C**). Graphical interface in FaceSync toolbox to manually align two audios. One audio file can be shifted in time using the sliders until the two waveforms are aligned. Alignment can be inspected visually by examining the waveform plots and by listening to the combined audio.

To facilitate the synchronization of videos, we developed FaceSync, an open-source Python toolbox, to automatically synchronize video recordings based on audio. In stimulus-based experiments, it requires a short audio segment to be played at the beginning of the experiment, which is recorded by the camera. Based on this shared audio, the toolbox can align the video to the beginning of the experiment finding the optimal alignment with the original audio segment. In unstructured social interaction experiments, multiple videos can also be aligned to a target video. The toolbox offers a sliding window search to find the offset that maximizes the correlation between two audio signals ( Figure 2) and a fourier transform based cross-correlation method. The FaceSync toolbox supports both Python versions 2 and 3 can be downloaded from our github repository ( https://github.com/cosanlab/facesync).

## Proof of concept validation of head-mounted cameras

In the following sections, we demonstrate that the head-mounted camera recording system provides a robust way to record facial behavior in laboratory experiments. In Study 1, we show that the head-mounted cameras can reliably record facial behaviors invariant to head rotation. In Study 2–4, we demonstrate the flexibility of using head-mounted cameras in multiple experimental setups including an event-based paradigm (Study 2), naturalistic video watching paradigm (Study 3), and a social interaction paradigm (Study 4). In these three proof of concept experiments, we also compare the performance of the FaceSync software in synchronizing recordings in comparison to the manual alignment method.

### Reliability of face recordings using head-mounted cameras

*Study 1: Face detection with head rotation.*

Methods. To examine the impact of head rotation on face registration, we recorded the face of one male participant (author J.H.C.) using a webcam and our head-mounted camera. In each recording session, the participant rotated the head 90 degrees left, returned to center, 90 degrees to the right, then returned to center. The head-mounted camera used a GoPro Hero4 camera to record 1280 x 720 resolution videos at 30 frames per second (fps). The webcam recording used the integrated camera on a Macbook Pro Retina laptop at 1080 x 720 resolution at approximately 30fps. Facial expressions were extracted using the iMotions Emotient FACET engine ( *iMotions Biometric Research Platform 6.0*, 2016) ^4^, which provides face registration success, landmark positions, AU predictions, and emotion predictions.

**Figure 3.**
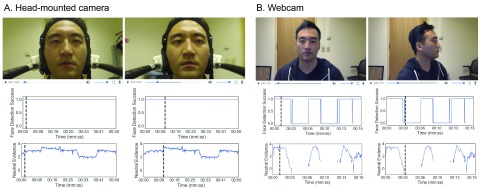
Face detection reliability between a head-mounted camera and webcam setup. ( **A**, **B**) Top row images show the face recordings when facing forward and the right panel image shows the face when facing left. Graphs in the middle row show face detection success at each frame. Graphs in the bottom row shows neutral face expression predictions. In ( **A**), facial expressions are predicted in all frames in contrast to ( **B**) where face detection and facial expressions predictions fail when the head is rotated away from the camera.

Results. Face recording using the head-mounted camera retained a continuous view of the entire face without face detection failure regardless of face rotation ( Figure 3A top row). In contrast, the webcam face recording resulted in face detection failure when the head was rotated ( Figure 3B right panel) which subsequently resulted in failure to extract facial expression predictions.

Face detection success was 100% of the video duration in the head-mounted camera recording compared to 75% in the webcam recording due to face detection failure when the face was turned ( Figure 3A, B middle row). Facial expression predictions (e.g., neutral face, Figure 3A, B bottom row) in the webcam recording also failed for 25% of the video during the head rotation while prediction from the head-mounted camera recording was unaffected.

Discussion. Comparing face recordings from a webcam and a head-mounted camera, we demonstrate that the head-mounted camera provides a more reliable and continuous recording of the face invariant to head rotation. This is important as face expression software is unable to make predictions about facial expressions when it is unable to register a face. Although the head-mounted camera is invariant to head rotations, the head position is currently not tracked. Future work might add additional sensors to monitor head position dynamics. Overall, we demonstrate that the head-mounted camera can prevent data loss due to body and head rotations that can readily occur in most experimental settings without strict restriction of participants’ natural movements.

### Flexibility of head-mounted cameras across multiple experimental settings

*Study 2: Recording facial expressions to event-based stimuli*

This experiment demonstrates the use of head mounted cameras in recording facial expressions to time-locked stimulus presentations. Performance of automatic video alignment using FaceSync in comparison to manual adjustment is also provided.

Methods. One male participant (author J.H.C.) viewed 10 positive images (e.g., kittens, puppies) and 10 negative images (e.g., injured bodies and faces) presented in MATLAB using Psychtoolbox version 3.0.12 ( Brainard, 1997; Pelli, 1997) and made deliberate facial expressions concordant with the valence of the image. Each image was presented for two seconds with jittered inter-trial intervals (ITI) of 4, 6, and 8 seconds (mean ITI = 5.4 seconds). The 20 images were selected from the IAPS picture database ( Lang *et al.*, 2008). Facial behavior was recorded using a head-mounted GoPro Hero4 camera in 1,280 × 720 resolution at 30fps.

Audio offset was determined both by manually synchronizing the recording using the FaceSync AudioAlign graphical interface and automatically using the FaceSync alignment function. The audio sample used for synchronization (synctune.wav) was a two-second harmonic tune constructed with sine waves at different frequencies. Four independent raters (including author J.H.C.) incrementally shifted the extracted audio in 1ms precision to the target audio using AudioAlign while listening to the shifted sound to minimize echo artifacts as well as visually checking the two waveforms for misalignment. For automatic alignment, we used the FaceSync sliding window correlation function (find_offset_corr) to detect the offset that maximizes the correlation similarity between the two audios. Alignment results from additional test videos that are not included in the face expression analysis were also tested for alignment and are reported as supplementary tests in Table 2. The video was trimmed according to the calculated offsets and facial expressions were extracted using the iMotions Emotient FACET engine ( *iMotions Biometric Research Platform 6.0*, 2016).

**Table 2.** Audio alignment results for study 2 – 4.

Study	Audio file	Target audio	Offset measured by manual adjustment				Offset measured automatically with FaceSync	Average difference between automatic and manual alignment (SD)
			Rater 1	Rater 2	Rater 3	Rater 4		
**Study 2**	Study2.wav	synctune.wav	7.921	7.920	7.913	7.917	7.916	-.002 (.004)
**Study 3**	s01_w.wav	bigbunny.wav	5.870	5.834	5.816	5.808	5.807	-.025 (.028)
	s02_w.wav	bigbunny.wav	5.401	5.377	5.399	5.387	5.387	-.004 (.011)
	s03_w.wav	bigbunny.wav	6.381	6.315	6.363	6.377	6.372	.013 (.030)
	s04_w.wav	bigbunny.wav	5.500	5.515	5.500	5.511	5.516	.010 (.008)
	s05_w.wav	bigbunny.wav	11.834	11.906	11.833	11.832	11.833	-.018 (.037)
**Study 4**	s02_d.wav	s01_d.wav	4.840	4.864	4.900	4.901	4.897	.021 (.030)
	s02_d.wav	s01_d.wav	5.930	5.973	5.983	5.959	5.978	.017 (.023)
	s04_w.wav	s01_d.wav	5.600	5.675	5.658	5.665	5.660	.011 (.034)
	s05_w.wav	s01_d.wav	4.502	4.507	4.508	4.511	4.504	-.003 (.004)
**Supplementary** **tests**	01.wav	clap.wav	18.922	18.924	18.924	18.920	18.924	.001 (.002)
	02.wav	clap.wav	16.216	16.218	16.218	16.217	16.218	.001 (.001)
	03.wav	synctune.wav	12.001	12.011	12.006	12.003	12.003	-.002 (.004)
**Overall mean difference**								.001 (.022)

Audio synchronization performance comparison between manual alignment and automatic alignment with FaceSync algorithm. Offsets indicate the duration in seconds from the original file that needs to be trimmed to be aligned to the target audio. One standard deviation shown in parentheses. Supplementary tests include audios from additional videos recorded for testing alignment, but are not part of Studies 2 to 4.

**Figure 4.**
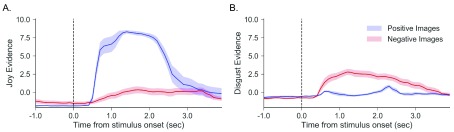
Different facial expressions for positive and negative images. ( **A**) Evidence of smiling to positive (blue) and negative (red) images. ( **B**) Evidence of disgust facial expression to positive (blue) and negative (red) images. Shaded error bars indicate standard error of the mean.

Results. An independent-samples t-test was conducted to compare positive facial expressions while viewing positive and negative images. Evidence of positive facial expressions while viewing positive images (M = 8.55, SD = .52) was significantly greater than the evidence while viewing negative images (M = 0.59, SD =1.53; t(18) = 15.58, p < 0.001; Figure 4A). Evidence for disgust facial expression while viewing negative images (M = 3.02, SD = 1.41) was significantly greater than the evidence while viewing positive images (M = .25, SD = .77, t(18) = 5.45, p < 0.001, Figure 4B).

Audio alignment results are reported in Table 2. The average difference between manual audio alignment by four different raters and the FaceSync automatic algorithmic alignment was -.002 seconds (SD = .004).

Discussion. In Study 2, we demonstrate the feasibility of using head-mounted cameras to record facial expressions in response to time-locked stimuli. The participant displayed positive facial expressions (i.e. smiling) to positive images and disgust facial expressions to negative images which was accurately retrieved from the analysis. Facial expressions were successfully linked to the stimuli that elicited the response by accurate alignment of the face recording to stimulus timing. Only a small difference was observed between the offsets determined automatically and manually. This study demonstrates the feasibility of using a head-mounted camera setup for standard computer-based experiments and that facial expressions can be linked with the stimuli that elicited the response with high temporal precision.

*Study 3 and 4: Watching and discussing naturalistic stimuli together*

To demonstrate the feasibility of using the head-mounted camera setup to simultaneously record facial expressions from several individuals, we recorded individual facial expressions of a group while they watched a video together in Study 3. Subsequently in Study 4, we recorded facial expressions of the group members while they discussed the contents of the video. We compare the facial expression behavior of each participant to one another with the expectation that participants would show synchronization of facial expressions in both conditions but to a greater extent in the movie watching experiment. Audio alignment offsets determined by manual and automatic alignment are compared.

Method. In Study 3, we measured the facial expressions of a group (N=5, 20% Female) watching a video, Big Buck Bunny ( “Big Buck Bunny,” 2008). In Study 4, the group freely discussed the content of the video. Each person’s facial behavior was recorded by their head-mounted GoPro Hero4 camera at 120fps and at 1,920 x 1,080 resolution.

In the Study 3, each face recording was aligned to the audio of the movie. In Study 4, each face recording was aligned to the audio of a single participant whose recording began the latest. In both studies, all cameras recorded the audio in the environment simultaneously, which allowed them to be aligned based on the shared audio. Each recording was aligned manually by four independent raters (including author J.H.C.) using the FaceSync AudioAlign graphical interface and automatically using the sliding window correlation alignment function (find_offset_corr). Differences in offset measured by the two methods were submitted to a one sample t-test to assess whether there was any difference between the two methods. After alignment, videos were trimmed using the FaceSync trim function.

Facial expressions, including AU activations and emotion predictions, were extracted using the iMotions Emotient FACET engine ( *iMotions Biometric Research Platform 6.0*, 2016) and subsequently downsampled to 1hz. To assess the similarity of the affective experience, we calculated intersubject synchrony (ISC). This technique has been used in fMRI analysis to identify signals that are common across participants when watching naturalistic stimuli such as movies and listening to stories ( Hasson *et al.*, 2004; Honey *et al.*, 2012; Nummenmaa *et al.*, 2012; Stephens *et al.*, 2010). For each experiment, ISC for joy facial expressions was calculated using pairwise correlation similarity of participants’ predicted joy time-series. To determine whether the group was synchronizing in their smiling greater than chance, we calculated a one-sample t-test over all pairwise correlations to test whether the synchronization was significantly different from zero. In addition, we used a paired-sample t-test to assess whether the ISC was significantly different between the viewing (Study 3) and the discussion (Study 4). All t-tests were conducted on Fisher r-to-z transformed correlations.

**Figure 5.**
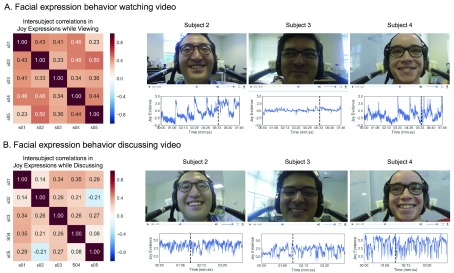
Facial expression behavior while watching and discussing video. ( **A**) Pairwise intersubject similarity matrix for joy while watching the video is shown on the left panel. Three subject videos are shown on the right with corresponding joy evidence predictions. ( **B**) Pairwise intersubject similarity matrix for joy while discussing the video is shown on the left panel.

Results. Overall, we found evidence that participants were having a similar affective experience while watching the video ( Figure 5). Average ISC was r = .40 (SD=0.08), t(9) = 13.89, p < 0.001. Participants also displayed synchronized facial expressions while discussing the video, r = .20, (SD=.16), t(9) = 3.75, p = 0.004. However, there appeared to be greater ISC of the joy facial expression viewing the show compared to discussing it afterwards, t(18) = 3.53, p = 0.002. This is likely because the joy facial expression was noisier while participants were talking and participants were not always in agreement with each other.

The average difference between the automated offset detection and the manual offset search was -.005 seconds (SD = .027; Table 2) for the movie watching videos and .011 seconds (SD = .024; Table 2) for the movie discussion session. Across all three studies and including additional supplementary videos that were similarly evaluated (see Table 2 footnote), overall differences in offsets calculated manually and automatically was .001 seconds (SD = .022) and was not significantly different (t(50) = .46, p = .65).

Discussion. In these two studies, we observed a relatively high level of synchronization in affective experiences across participants while viewing and subsequently discussing a video. Speaking appears to decrease the sensitivity of ISC, likely as a result of the added noise from the mouth movements while speaking. The FaceSync toolbox can accurately align videos together even when the audio recordings are non-uniform due to location of the camera position and multiple people talking at the same time. Overall, these studies demonstrate the flexibility of using head-mounted cameras to record facial behavior in naturalistic experimental paradigms such as watching a movie and also in social experiments, in which participants interact with each other.

## Discussion

In this paper, we provide evidence that head-mounted cameras offer a robust, flexible, and affordable solution to recording facial expressions in the laboratory. In four proof of concept studies, we first demonstrate that the head-mounted camera yields *reliable* face recordings that allow facial expression analysis irrespective of head motion. Second, we demonstrate the flexibility of using head-mounted cameras across *different experimental settings* from traditional stimulus based experiments to group social interaction experiments. By using the FaceSync toolbox to align recordings to stimulus onsets, facial expressions were successfully linked to the events that triggered facial behavior, such as increased positive facial expressions in response to viewing positive images, and increased disgust facial expressions in response to viewing negative images. In addition, facial expressions were more synchronized when watching a video compared to when discussing the video. Most importantly, we demonstrate that the FaceSync toolbox can accurately and automatically align video recordings with comparable accuracy with manual alignment. Together, these results demonstrate that the head-mounted camera setup offers a reliable and robust method for recording facial expressions in a variety of experimental settings and can scale to n-person social experiments.

The head-mounted camera setup can still be improved in several ways. For example, lighting conditions are important in face detection such that poor luminance of the face or extreme backlights can lead to face detection failures. Researchers should be aware of this issue and should avoid situations where ceiling lights or window sunlight in the background decrease face luminance. LED lights can be attached to the head mount to control for these issues by providing equal luminance of the face.

Another potential improvement is the weight and size of the camera. The weight of the camera that pulls the headgear downward can be a source of discomfort if worn for extended periods of time. At the time of construction, the camera (i.e., GoPro Hero4 Black with camera, memory card, and lens cover) weighed 92 grams but now more recent models (i.e., GoPro Hero5 Session) weigh only 74 grams. Small reductions in weight can lead to increased comfort as the tugging force is significantly reduced based on the length of the headset. Researchers can take advantage of newer and lighter cameras as they become available as the 3D printable camera mount provided with the blueprints is compatible with other cameras.

In summary, we hope that these tools can benefit other researchers and further accelerate facial expression research. We anticipate that this framework will aid in improving our understanding of how emotions are experienced in naturalistic settings and how emotions surface and influence social interactions. In addition, we hope that these tools can aid researchers in developing new models of complex emotions such as regret, guilt, and gratitude. We look forward to a new era of facial expression research that examines facial behavior in both controlled and naturalistic settings to yield a robust and holistic understanding of how facial expressions reflect our thoughts, emotions, and future behavior.

## Ethical statement

This study was approved by the Institutional Review Board at Dartmouth College and written consent from all participants was obtained. Informed written consent was obtained for all individuals whose full faces are shown in the figures or in the videos in data repository.

## Software availability

Source code for FaceSync toolbox available from: https://github.com/cosanlab/facesync

Archived source code at time of publication: https://doi.org/10.5281/zenodo.2638335 ( Cheong *et al.*, 2019)

License: MIT

## Data availability

### Underlying data

Open Science Framework: FaceSync: Open source framework for recording facial expressions with head-mounted cameras. https://doi.org/10.17605/OSF.IO/B2NUA ( Cheong *et al.*, 2019)

This project contains the following underlying data:

- Analyses folder containing: FiguresForFaceSync_OSF.ipynb (Jupyter Notebook with scripts to completely recreate the analyses and figures)
- Data folder containing: Raw data necessary to recreate the analyses, such as videos, extracted facial expression information, and sounds used for synchronizing the videos (in .txt, .csv, .wav and .MP4 formats)
- Supplementary Information folder containing: FaceSync_build_information.pdf (detailed instructions on how to build the FaceSync head-mounted recording system, including parts List and build instructions, and Supplementary Tables 1 and 2)
- Data are available under the terms of the Creative Commons Attribution 4.0 International license (CC-BY 4.0).

## Footnotes

^1^Number of articles retrieved from Pubmed Central search with keyword ‘facial expressions’.

^2^Quote retrieved from http://facewaretech.com/pricing/ on July 2017.

^3^Cost estimated at time when manuscript written on July 2017.

^4^Alternative free software such as OpenFace ( Baltrusaitis *et al.*, 2018) can also be used to replicate the study.
