# Supplementary material for: FaceSync: Open source framework for recording facial expressions with head-mounted cameras
Source: F1000Res. 2019 May 21;8:702. [Version 1] doi: 10.12688/f1000research.18187.1 (PMC7059847; doi:10.12688/f1000research.18187.1)
Supplement: Peer review history

# Peer review: Reviewer response for version 1

**DOI:** 10.5256/f1000research.19894.r60016

**Published:** 2020-03-05

**Authors:** Sylwia Hyniewska

**Affiliations:** 1 Psychology and Language Sciences, University College London, London, UK

The article describes head-mounted camera systems as the most appropriate state-of-the-art recording devices for facial expression analysis. Two drawbacks of such setups are emphasised by the authors: the cost of commercialised headmounts and synchronisation of recordings with studied stimuli.

1. First, the authors provide step-by-step assembly instructions to enable researchers to easily construct their own head-mounted camera setup as an affordable alternative option that requires minimal engineering expertise.
2. Second, the authors introduce FaceSync, an open-source Python toolbox that they developed to automatically synchronize video recordings based on audio cues. A short audio segment can be played at the beginning of the experiment by e.g. a stimuli presenting software and be recorded by the head mounted camera. The toolbox allows the alignment of the video to the sound produced by the experimental software. In unstructured social interaction recordings, multiple videos can be aligned to a target video. The toolbox offers a sliding window search to find the offset that maximises the correlation between two audio signals and a fourier transform based cross-correlation method. To test that the head-mounted camera recording system provides a robust way to record facial expressions, the authors ran several studies with users.

 

 Nowadays, however, facial expression analysis is not limited to fixed cameras and webcams. The authors describe perfectly the added value of head mounted cameras for (automatic) facial expression analysis but do not describe the current state of the art in terms of comparable hardware and software. Given fast progress, e.g. in immersive technologies, a variety of solutions exist. The authors should consider discussing the expensive commercial options (e.g. Google glasses with Emotiv SDK and OpenCV for visual processing) and the open access options that do exist, e.g. for video alignment and facial expression recording from similar devices. If no user study can be done on similar hardware and software for comparison, please provide sufficient information in the state of the art and discussion sections.

 Please consider emphasising the added value of the article/framework in the title and abstract.

 Please make it clearer whether FaceSync is a synchronisation system (based on audio) as described in the manuscript or a framework for recording facial expression, as in the title.

I confirm that I have read this submission and believe that I have an appropriate level of expertise to confirm that it is of an acceptable scientific standard.

# Peer review: Reviewer response for version 1

**DOI:** 10.5256/f1000research.19894.r52689

**Published:** 2019-09-10

**Authors:** Elliott Ross

**Affiliations:** 1 Department of Neurology, University of Oklahoma Health Sciences Center, Oklahoma City, USA

This is a “proof of concept” publication that introduces a novel head-mounted camera apparatus for obtaining spatially accurate videos of facial expressions that is able to compensate for unwanted head movements that often occur during research projects, especially those that involve spontaneous facial expressions. The apparatus uses a small, light-weight and relatively inexpensive Go-Pro video camera (30 fps, 1,280 x 720 pixels) that is mounted on a rectangular(light-weight frame work that in turn has a head mount that rests on top of the head and over the ears so that it does not block or interfere with the facial muscles that give rise to forehead expressions (see Figure 1, panels D and C, and Figure 3, panel A). 

 The authors provide directions for construction of the apparatus with blueprints and provide a means for the facial expression videos to be synchronized with audio recordings for accurate off-line analyses (FaceSync). In addition, the authors present 4 brief “proof of concept” experiments that demonstrate the utility of their apparatus and FaceSync. The most important is Study 1, in which the subject moves his head 90 degrees to the left and then 90 degrees to the right of center. The video was then analyzed off-line using the iMotion Emotient FACET engine that provides “face registration success, landmark positions, AU predictions and emotion predictions.” Face detection was 100% successful for the entire video. In comparison, using a webcam (stationary) video recording for the same sequence, only 75% of the video was successfully analyzed because the iMotion Emotient FACET engine failed to detect the face during head rotation.

 This publication makes a major contribution to the quantitative analysis of facial expressions that use video recordings. To date, research videos of facial expressions are obtained by using a fixed camera set-up that cannot compensate for spontaneous head movements, i.e. a computer webcam, a tripod-mounted camera or a pan-tilt-zoom camera (see Figure 1, panels A, B and C). Although software exists that can partially compensate for head movement in a given plane, it is mathematically very difficult, if not impossible, to compensate for head movements that occur over multiple planes, especially rotatory head movements, when attempting to quantitatively analyze facial expressions using computer software techniques. Thus, the head-mounted camera apparatus will allow researchers to obtain better and more complete quantitative data regarding facial expressions. My only suggestion to the authors is that they might consider adding an adjustable counter weight to the back of their apparatus to make the frame more comfortable for the subject.

I confirm that I have read this submission and believe that I have an appropriate level of expertise to confirm that it is of an acceptable scientific standard.
